# The Only Pebble on the Beach: Dr. Archie’s Little Joke

**Published:** 1901-07

**Authors:** 


THE ONLY PEBBLE ON THE BEACH: DR. ARCHIE'S LITTLE JOKE.—The *Detroit Medical Journal* is three months old, going on four. It is edited by Dr. G. Archie Stockwell. It has printed on its front cover page the claim that it is "the only strictly ethical medical journal published in America." I have received Vol. I, No. 3, marked "sample copy." I immediately put it on ice. Anything so young, so tender and so pure should be protected from the summer heat, which, at Austin, is up in the nineties; it's apt to sp'ile.

I had thought to ask Dr. Archie to exchange, but a moment's reflection showed me that he "das'nt"; it would be "unethical" to exchange with a hoary old sinner like the *Red Back*, or with Roberts or Simmons, or Culbertson or Love, or Emmett or any other of the unethical fellows. So, Dr. Archie, being the whole thing, will have to "be good and be lonesome." I suggest that he add to the announcement on his cover page, "Oh, Lord, I thank thee that I am not like old Dan'els, Love, Roberts and Culbertson and those other old publicans and sinners, being more holy than they; likewise more ethical."

The Doctor ought to bear in mind that the good die young, and look a little out.

Now, I was at first inclined to take Dr. Archie seriously. On reflection I am satisfied that he doesn't mean it. It is only one of his little jokes. He *can't* mean it! Now, here am I, taught at Sunday school when I was in knickerbockers that to be virtuous is to be happy; and that virtue has its own reward,—I, who have tried *so hard* all these years to be "virtuous," to be "ethical," and have been looking every day for my reward to come by mail. Are those efforts all to go for nothing,—my hopes to be dashed in any such way? Am I to be denounced by young Archie as "unethical" in my old age? Surely not. Dr. Archie is joking. I fear he is a terrible wag.

Meantime, I'm keeping the pure little thing on ice.

